# Left Out and At Risk: Post-Pandemic Continuation of Organizational Service Reduction in Metropolitan New York City Coincides with Rise in Opiate Use and Mental Health Problems for Latinos

**DOI:** 10.3390/ijerph23050628

**Published:** 2026-05-08

**Authors:** Ruth Lin Campbell, Smita Ekka Dewan

**Affiliations:** 1 Independent Researcher, P.O. Box 1235, New Paltz, NY 12561, USA; 2 Human Services Department, New York City College of Technology, City University of New York, 300 Jay St., Brooklyn, NY 11201, USA; sdewan@citytech.cuny.edu

**Keywords:** Latino, organizational facilitation, opiate use, mental health, prevention

## Abstract

**Highlights:**

**Public health relevance—How does this work relate to a public health issue?**

- This study documents provider-reported declines in organizational support for Latino substance use help-seeking in New York City (2018–2024) and persistently low mental health support for Latinos statewide during the post-pandemic period (2022–2024), both of which predict poorer health outcomes.
- New York State (NYS) is one of the states with a higher Latino population density, so this NYS provider report may be relevant to other states with similar rising mental health and substance use problems among urban Latinos.

**Public health significance—Why is this work of significance to public health?**

- Predicting poor outcomes for NYS Latinos amid rising substance use and mental health issues highlights a critical need for targeted treatment improvements.
- The deterioration of organizational resources for substance use and mental health treatment in domains of language accommodation, financial and insurance support, cultural responsiveness, and physical access signifies avenues for intervention and treatment research at policy, community, organization and individual levels.

**Public health implications—What are the key implications or messages for practitioners, policy makers and/or researchers in public health?**

- Implications underscore the importance of targeted outreach and resource linkage at all levels for younger and older urban Latinos to improve health outcomes.
- Implications for practitioners, policy makers and researchers suggest a focus on the improvement of Latino treatment services in specific organizational domains to improve health outcomes.

**Abstract:**

Increasing opiate use and mental health problems among younger and older Latinos in urban US contexts prompted this investigation into the organizational resources that facilitate help-seeking efforts of Latinos in New York State (NYS). Guided by a Vulnerability Model and a framework of organizational enabling resources, this study used complementary longitudinal and cross-sectional designs. The longitudinal component examined changes in levels of organizational facilitators for Latino substance use disorder (SUD) help-seeking in Downstate NYS across three time periods, while the cross-sectional component compared post-pandemic facilitator levels across service types and regions. A convenience sample of 241 SUD clinicians participated in the longitudinal analysis. A sample of 150 clinicians whose practice information varied by location and service type participated in the post-pandemic cross-sectional comparisons. The findings revealed that the proportion of clinicians reporting organizational facilitation of Latino SUD help-seeking in Downstate NYS has diminished significantly from pre-pandemic levels, with little recovery. Cross-sectional analyses revealed no significant differences in clinician estimation of post-pandemic organizational resources by region or service type. The findings suggest that the organizational bulwarks against SUD and mental health problems are not sufficient to mitigate public health risk for NYS Latinos in the post-pandemic period. Recommendations informed by provider perspectives are discussed.

## 1. Introduction

Vulnerable communities struggle to maintain integrity and coherence in times of social change. During times of political upheaval and health crises, communities such as those of NYS Latinos may lose access to vital healthcare. This paper investigates the levels of organizational facilitators for the SUD help-seeking efforts of Latinos in Downstate NYS from the pre-pandemic period through the pandemic period into the post-pandemic period using a longitudinal analysis with a cohort of 241 NYS licensed SUD clinicians. It then uses a cross-sectional analysis with a sample of 150 NYS clinicians with varied practice information to compare reports of post-pandemic organizational facilitation for Latino help-seeking by location (Downstate NYS vs. Upstate NYS) and service type (SUD vs. non-SUD) throughout the state.

A comprehensive individual and community-based model of vulnerability and a framework of organizational enabling resources in four domains (language accommodation, legal/financial/insurance support, cultural responsiveness, and access to services) guided the investigation of this aspect of Latino public health.

### 1.1. Rising Latino Healthcare Needs

#### 1.1.1. Rising Opiate Use

NYS Latino communities currently face the challenge of rising opiate use. According to reports from the National Survey on Drug Use and Health [1], Latino youth, concentrated in urban areas such as New York City’s center, are finding easy access to heroin and have a diminished risk perception of the harm of heroin use [2]. For 12- to 17-year-old Latino youth, opioid use disorders have increased significantly over the past year [3]. For Latino young adults (18–25 years old) who are unemployed, prescription opioid use is higher [4]. Latino youth and young adults with no insurance coverage and those who are poor are most at risk. Rising gang violence among young Latinos in metropolitan areas such as the NYC center accompanies rising opiate use [5].

The combination of potential risk factors, including US nativity, place many in this group at higher risk [6].

Nationally, only 2.9% of Latinos who used opiates in the past year received any substance use disorder (SUD) treatment [7].

#### 1.1.2. Rising Mental Health Needs

Mental health needs increased for Latinos over 26 years of age, especially for those residing in cities such as NYC, and especially for those with no insurance coverage [8]. Nationwide, 44.1% of Latinos [7] met the criteria for any mental illness in 2024, while only 16.4% of Latinos received treatment for mental health needs [9].

### 1.2. A Comprehensive Vulnerability Framework

**Figure 1:**
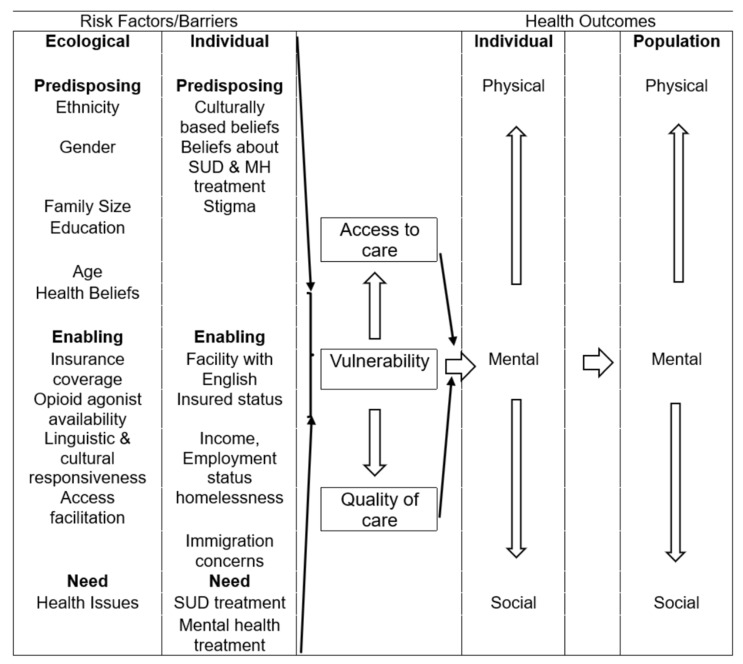
Vulnerability model outline of predisposing, enabling and need factors.

Vulnerable communities, such as the NYS Latino community, demonstrate unique environmental and individual risk and protective factors that interact to create barriers or to facilitate health outcomes. A Vulnerability Model [10] is tailored in Figure 1 to illustrate factors that affect the service use of Latino community members. Used and modified with the permission of the authors, this model presents a picture of interacting environmental and individual factors that influence health, demonstrating how predisposing, enabling, and need factors interact to cumulatively result in health outcomes for individuals. Individual predisposing, enabling and need factors are outlined below; however, this paper focuses most closely on organizational (see the Ecological category in the Vulnerability Model below) enabling factors—those which are under the control of organizations—which may enable help-seeking Latinos to receive services.

#### 1.2.1. Individual Factors

For Latino community members, individual predisposing factors incline them to use available health care resources and include health beliefs about the stigma of mental health and substance use disorders [11,12,13], cultural beliefs [13,14,15], and beliefs about treatment [11,16,17]. Individual enabling factors incline an individual toward or away from the use of services, and include facility in English [16,18], homelessness and poverty [19,20], employment status [18,19], insured status [11,15,20,21] and documentation/immigration issues [13,16]. Need factors for this population include help with SUDs and mental health problems [11,20,22,23], need for translation or language facilitation [24], and the need for help with the presence of chronic health conditions [18].

#### 1.2.2. Organizational Factors

Organizational enabling factors may include insurance coverage for services [25,26], access facilitation [27], the availability of opioid agonist therapy [19] and linguistic and cultural responsiveness [13,28].

This paper examines SUD organizational facilitation of Latino help-seeking across three time periods, comparing the availability of resources in four domains in the pandemic wave (2020–2022) and the post-pandemic wave (2022–2024) to the availability in the pre-pandemic period (2018–2020). In the post-pandemic period, we broadened our exploration of organizational facilitation of Latino help-seeking by location and organizational service type to include Upstate NYS and non-SUD organizations.

### 1.3. Problem Statement and Hypotheses

To investigate the organizational facilitation patterns of NYS organizations, we developed the following hypotheses:

**Hypothesis** **1.** *From the pre-pandemic wave (P1) to the pandemic wave (P2), the proportion of service providers in Downstate NYS reporting the presence of organizational facilitators for Latino SUD help-seeking will decrease significantly across four domains: (a) language accommodation, (b) legal/financial/insured support, (c) cultural responsiveness, and (d) access to services.*

**Hypothesis** **2.** *From the pandemic wave (P2) to the post-pandemic wave (P3), the proportion of service providers reporting the presence of organizational facilitators will not significantly increase across the four domains of (a) language accommodation, (b) legal/financial/insured support, (c) cultural responsiveness, and (d) access to services, indicating the persistence of pandemic-related reductions.*

**Hypothesis** **3.** *(Trajectory): Across the three waves (P1, P2, P3), organizational facilitators in the four domains of (a) language accommodation, (b) legal/financial/insured support, (c) cultural responsiveness, and (d) access to services, will exhibit a decline from P1 to P2 and remain significantly below pre-pandemic levels at P3.*

**Hypothesis** **4.** *In the post-pandemic wave (P3), the proportion of service providers reporting the presence of organizational facilitators for Latino help-seeking in NYS in four domains will differ by location (Upstate/Downstate), i.e., (a) language accommodation, (b) legal/financial/insured support, (c) cultural responsiveness, and (d) access to services.*

**Hypothesis** **5.** *In the post-pandemic wave (P3), the proportion of service providers reporting the presence of organizational facilitators for Latino help-seeking in NYS in four domains will differ significantly by the type of help sought (SUD/non-SUD), i.e., (a) language accommodation, (b) legal/financial/insured support, (c) cultural responsiveness, and (d) access to services.*

## 2. Materials and Methods

### 2.1. Participants

All NYS clinicians older than 21 years of age were emailed invitations to participate in a free six-credit (CEU) training opportunity in English and Spanish, which was part of a research study on how healthcare treatment is facilitated for Latinos. Completion of the study questionnaires was a requirement for the training. Study investigators used a sampling frame list of email addresses of active (approximately 6000) clinicians, certified by the Office of Addiction Services and Supports (NYSOASAS, Albany, NY, USA), to develop a non-probability sample of participants with the Credentialed Alcoholism and Substance Abuse Counselor (CASAC) certification. Additionally, we used a sampling frame list of over 40,000 email addresses of clinicians with three other specialties that was obtained by request to the NYS Education Department (Albany, NY, USA). The full sample included 43,000 NYS clinicians in social work, mental health counseling, marriage and family counseling and certified alcohol and substance use disorder specialties.

Additionally, a brief three-open-ended-question survey was sent out in October and November of 2025 to the 43,000 NYS clinicians listed above requesting their qualitative comments for the improvement of services for Latinos in New York State. This survey was designed as a contest that provided $100 prizes for 10 of those 168 clinicians who completed the survey.

Advertising methods included special notices on the NYSOASAS website and attempted phone contact with each of the directors of the NYSOASAS facilities to alert clinicians and clinic directors to the training opportunity. Digital advertisements were sent directly to the clinician email list that was provided via contact with the NYS Education Department. Those who registered signed informed consent, indicating that they understood that they were participating in the research study. Copies of the survey instruments are included in Supplementary Materials.

### 2.2. Exclusion Criteria for Hypothesis 1–3

Hypotheses 1, 2 and 3 focused on the organizational resources of Downstate NYS area participants who offered SUD treatment. The exclusion of providers from all other locations (Upstate) and of other types of services (non-SUD) from this analysis resulted in a sample of 241 participants in the dataset who all provided substance use treatment and lived in Downstate New York. The data from these participants stemmed from collection during the three waves of the study: pre-pandemic (*n* = 176, 73.03%), pandemic (*n* = 31, 12.86%), and post-pandemic (*n* = 34, 14.11%). Demographics and practice characteristics for this group are documented in the Results section.

### 2.3. IRB Approval

The ethical protection of research subjects and approval of research protocols were carried out by three IRB groups: Hummingbird Independent Review Board, the Western Copernicus Group Independent Research Board and the North Star Review Board.

### 2.4. Measures

The study collected data on independent variables of time period and location by Latino population density. Data were also collected on the dependent variable of organizational facilitators of Latino involvement, with four domains: language, legal/financial status, cultural, and geographical/access. Latino service utilization was measured by Caseload Percent in P1 and P2 and measured by Percentage Hours Delivered in P3 (see Limitations Section). Qualitative provider comments concerning the improvement of services for Latinos are included. Clinicians’ sociodemographic and practice information were collected for descriptive purposes.

#### 2.4.1. Independent Variables

Time Period. Three time periods (waves) were compared: 2018–2019 data were placed in the pre-pandemic category (P1), March 2020–April 2022 data were placed in the pandemic category (P2), and October 2023–July 2025 data were placed in the post-pandemic category (P3).

Location by Latino Population Density. Clinician responses were categorized into two groups, Upstate (county Latino population density ≤ 10%) and Downstate (county Latino population density > 10%) based on the Latino population density in the county of the clinician’s practice [29]. A list of NYS counties which were included in each region is included in Supplementary Materials.

#### 2.4.2. Dependent Variables

Organizational Facilitators (P1 and P2). Four domains capture the representation of each of the organizational facilitators: language, legal/financial status, cultural, and geographical/access. In the first two waves (P1 and P2), each domain was represented by four closed-ended questions and one open-ended question. Clinicians were invited to remark on the resources that might facilitate Latino utilization of treatment with a final open-ended question. For example, a representative question is “LEGAL & FINANCIAL: The resources listed below might help Latinos overcome legal or financial barriers in order to get help. Please check the ones that your organization (including your private practice, if any) offers”. The four closed-ended answer choices were: (a) a list of phone numbers for legal help in Spanish, (b) a paper or brochure in Spanish concerning the rights of undocumented Latinos, (c) instructions in Spanish to help Latino clients get access to coverage for services, or (d) none of the above. The open-ended invitation for comments used the following language: “If there are other ways in which your organization facilitates the Latino use of services through legal or financial resources, please explain below”.

Service Utilization—Caseload Percentage. This variable was represented by the percentage of Latinos in each SUD counselor caseload, as estimated by the counselor. These data were only collected for the P1 and P2 periods (See Limitations Section).

Service Utilization—Percentage of Hours Delivered. This variable was represented by the percentage of service hours that each active clinician delivered to Latino clients during the most recent two-week interval. These data were collected only for the P3 period (See Limitations Section).

Organizational Facilitators (P3). For the post-pandemic (P3) analysis, the four domains of language accommodation, legal/financial support, cultural responsiveness and geographical/access to services were retained, along with the four closed-ended questions and one open-ended question that elicited the description of the domain. However, research current with the P3 period identified expanded definitions of cultural responsiveness and access to services. We felt it would be remiss of the study to limit the measures of cultural responsiveness and access to our original formulation. We anticipated making the measures dichotomous to be more easily comparable to the earlier formulations. For that reason, in the P3 period part of the study, the subcategories of cultural responsiveness and access domains were expanded to include descriptive lists of characteristics that emerging sources had identified as important descriptors of the domain at the time of the study design.

Cultural Facilitators (P3). Questions were added to the original four-question measure in accordance with suggestions derived from materials developed by the Office of Minority Health, available in the public domain, and listed as Enhanced National Culturally and Linguistically Appropriate Services (CLAS) Standards. At the time of the post-pandemic wave (2022), it was available as the Agency Cultural Competence Checklist—Revised Form [30,31] and had been used to measure the cultural responsiveness of agencies with Hispanic American individuals. The 23-item checklist is available in the Results Section.

Access Facilitators (P3). The measure for access facilitators (P3) was expanded in accordance with the literature, which suggested that the distance and timeliness of services is an important factor in effective treatment [32,33]. For that reason, “immediate treatment,” “flexible hours”, “easy access”, and “location” were added to the three items that already existed in the questionnaire to measure the facilitation of geographical access, i.e., “telehealth”, “late hours”, and “transportation”.

#### 2.4.3. Qualitative Comments (P3)

Qualitative comments from providers on the improvement of Latino services in NYS were collected in October and November of 2025 via SurveyMonkey. One of the questions from this survey is “What might a program that is culturally relevant for Latinos be like? (Bilingual educational groups for the family member not in treatment? bi-lingual childcare for Latino children? telehealth? No telehealth? Is time of day important? Is anonymity important? Is transportation important?) Please express your ideas with a few sentences”. The questionnaire is available in the Supplementary Materials.

Other sociodemographic and practice measures were formulated as suggested by the research standards.

#### 2.4.4. Data Collection

Data collection was performed using questionnaires administered electronically via SurveyMonkey. Data were collected consistently throughout the years 2018–2024. The free six-CEU course was continually offered to NYS clinicians. When data were harvested in 2025, the pre-pandemic (P1) (2018–2019), pandemic (P2) (March 2020–2022) and post-pandemic (P3) (2023–2024) data were separated for analysis.

Data from participants was merged from the pre-pandemic (P1), pandemic (P2), and post-pandemic (P3) waves to create the final dataset. The method resulted in the collection of 357 questionnaires completed by NYS clinicians, which documented data on the variables of interest in the study. The collection of 168 questionnaires completed by NYS clinicians documenting their opinions concerning the improvement of services was performed separately during October and November of 2025.

#### 2.4.5. Analytic Strategy

A longitudinal design allowed for the comparison of the proportion of service providers’ reporting of organizational facilitators of SUD treatment for Latinos across the three time periods.

A bivariate analysis was conducted to test all research hypotheses. Depending on the level of measurement of the variables, Chi-square analyses and Pearson’s correlations were used to compare the variables across the three phases. Across analyses, the initial α-level was set to 0.05. However, the Šidák correction was applied consistently when multiple comparisons were performed to reduce the family-wise error rate.

Four Chi-squared (χ^2^) Tests of Association compared provider reports of SUD organizational facilitator data during the pre-pandemic (P1), pandemic (P2) and post-pandemic (P3) periods for the Downstate location. The outcome of this analysis tested the hypothesized differential proportion of service provider reports of SUD organizational facilitators from the pre-pandemic (P1) to the pandemic wave (P2) (Hypothesis 1a–d). The analysis tested the hypothesized differential proportion of service provider reports of SUD organizational facilitators from the pandemic (P2) to the post-pandemic wave (P3 (Hypothesis 2a–d). The analysis tested the hypothesized trajectory of the proportion of service provider reports of SUD organizational facilitators over the P1–P3 period (Hypothesis 3a–d).

Chi-square analyses for the post-pandemic (P3) data compared service provider reports of organizational facilitators in two locations—Downstate and Upstate—and for two types of treatment—SUD and non-SUD. The outcome of this analysis tested Hypotheses 4a–d and 5a–d concerning the comparison of the proportion of service provider reports of facilitators by location (Downstate vs. Upstate) and by type of service (non-SUD vs. SUD).

Service utilization by Latinos was not comparable over the three waves because of measurement issues. This will be discussed further in the Results and Limitations Sections.

## 3. Results

### 3.1. Downstate SUD Organizations

#### 3.1.1. Three-Wave Sociodemographic Comparison

As shown in Table 1, sampled clinicians who offered SUD treatment in the Downstate location (*n* = 241) ranged in age from 25 to 76 years, with an average age of 52. 8 years (*SD* = 11.1). Most participants identified as female (66.8%) and non-Hispanic Caucasian (43.2%) who had earned a graduate degree (51.5%) and the CASAC certification (87.6%). Most practiced in a private or outpatient practice setting (44.4%). Downstate SUD service providers in the pre-pandemic period reported a Latino caseload average of 23.8%, while those in the pandemic period reported a significantly lower caseload average of 15%. In the post-pandemic period, the proportion of hourly SUD services delivered to Latinos averaged 18.9% in the Downstate region.

Differences in the means and frequencies for demographic variables were tested using analysis of variance (ANOVA) and the Chi-squared (χ^2^) Test of Association. For the χ^2^ analyses, none of the analyses except for the CASAC certification status violated the assumption of expected cell counts below 5 (2/6 cells). In this regard, Fisher’s Exact Test was used instead. Using ANOVA and χ^2^, neither the age of participants nor the gender, nor the practice settings varied significantly by wave. In contrast, levels of education (*p* = 0.002), races/ethnicities (*p* < 0.001), and CASAC certification status (Fisher’s Exact Test, *p* < 0.001) did vary significantly by wave when using the χ^2^. Regarding these significant χ^2^ analyses, post hoc testing (adjusted Pearson residuals) was used to identify specific cells in which the observed cell differed from the expected cell. To account for multiple comparisons, the Šidák adjustment was used for race/ethnicity (12 comparisons; α_Šidák_ = 0.0043), education (nine comparisons; α_Šidák_ = 0.0068), and CASAC (six comparisons; α_Šidák_ = 0.0085).

In the pre-pandemic wave (P1), there were greater percentages of participants who identified as Black, with bachelor’s degrees, and with CASACs than expected. In the pandemic wave (P2), there were greater percentages of participants who identified as White. In the post-pandemic wave (P3), there were more participants who identified as Other, who had graduate degrees, and who did not hold CASACs.

#### 3.1.2. Three-Wave Organizational Facilitators Comparison

Descriptions of the language, legal/financial, cultural and access resources reported by Downstate SUD clinicians across the three waves are listed in Table 2.

All four of the χ^2^ analyses of resources were statistically significant: Language, Fisher Exact Test, *p* < 0.001, *V* = 0.28; Legal/Financial, χ^2^(2, *N* = 241) = 38.86, *p* < 0.001, *V* = 0.40; Cultural, χ^2^(2, *N* = 241) = 51.72, *p* < 0.001, *V* = 0.46; and Access, χ^2^(2, *N* = 241) = 16.34, *p* < 0.001, *V* = 0.26. Note that one cell (1/12) had an expected value below 5 for Language, prompting the use of the Fisher Exact Test (as reported above). Given the significant χ^2^ tests, post hoc testing using Šidák-adjusted α-levels followed. Follow-up pairwise χ^2^ tests (2 × 2 contingency tables) were conducted to analyze the three *a priori* pairs (P1–P2, P2–P3, P1–P3) shown in Table 3. As there were three comparisons, α_Šidák_ = 0.017.

Three-Wave Language Facilitators Comparison. As shown in Table 3, prior to the pandemic, 90.3% of the surveyed Downstate NYS clinicians reported that their clinics had language resources to facilitate Latino client involvement in treatment. Clinics and clinicians had developed creative methods to overcome language barriers. As shown in Table 2, Downstate clinicians most commonly (38.6%) mentioned Spanish-speaking clinicians who met the needs of Spanish-speaking clients. Providers with enough Spanish-speaking clinicians matched the Spanish-speaking clientele with those clinicians. In comments from the open-ended questions which accompanied the closed-ended survey questions, providers with more Spanish-speaking clients than their clinics could easily serve noted that they used strategies that increased the client-to-provider ratio. For instance, they referred Latinos who could not speak English well to “resources that can assist them in their language,” “ran groups in Spanish”, used adjunct staff, or used technical assistance, such as “phone translation”, “CRYACOM”, translator lines or automatic translators.

As shown in Table 3, the percentage of SUD clinicians reporting language resources dropped dramatically and significantly during the pandemic wave from 90.3% to 64.5% (P1–P2 comparison: χ^2^(1) = 15.03, *p* < 0.001). The post-pandemic wave did not demonstrate any significant recovery in language resources, shown by the P1–P3 comparison, which continued to demonstrate significant difference (did not survive the corrected α-level) [χ^2^(1) = 9.92, *p* = 0.002], while the P2–P3 comparison was non-significant [χ^2^(1) = 0.27, *p* = 0.60].

Three-Wave Legal/Financial Facilitators Comparison. As shown in Table 3, prior to the pandemic, 86.4% of the surveyed Downstate clinicians reported that their clinics facilitated Latino client involvement in treatment by providing legal/financial information. This included information on legal help, undocumented rights and insurance coverage. As shown in Table 3, the percentage of SUD clinicians reporting resources dropped dramatically and significantly during the pandemic from the pre-pandemic level of 86.4% to 48.4% [χ^2^(1) = 24.38, *p* < 0.001]. The slight decrease to 47.1% of clinicians in the post-pandemic (P3) wave reporting that their clinics provided such resources for Latinos was not statistically significant (P2–P3 comparison: χ^2^(1) = 0.01, *p* = 0.92).

Three-Wave Cultural Facilitators Comparison. As shown in Table 3, before the pandemic, the majority (82.4%) of the surveyed Downstate SUD clinicians reported that their clinics facilitated Latino involvement by means of cultural resources, which included a list of phone numbers and outreach at churches. Downstate clinicians (22.7%) cited a list of phone numbers for welcoming Latinos to local neighborhoods as the most commonly provided clinic cultural resource. Downstate clinicians (15.3%) reported that their clinics provided outreach to churches or festivals that Latinos might attend.

As shown in Table 3, Downstate SUD clinicians reported significant decreases in cultural facilitators from pre-pandemic levels (82.4%) to pandemic levels (54.8%) [χ^2^(1) = 11.76, *p* = 0.001] and from pandemic to post-pandemic levels (23.5%) [χ^2^(1) = 6.72, *p* = 0.01]. Chi-square comparisons were statistically significant across all three time periods, indicating significant decreases in clinician reports of organizational cultural resources between and across time periods.

Three-Wave Access Facilitators Comparison. As shown in Table 3, the majority (84.7%) of the surveyed SUD clinicians in the pre-pandemic period reported that their clinics had resources that could facilitate Latino access and involvement. In open-ended comments, clinicians reported that their clinics “provided Metro cards to clients who needed transportation”, and “were open during evening hours” for those who worked. Although clinicians reported that telehealth resources continued to increase over the three periods, from 8% in the pre-pandemic wave to 38.2% in the post-pandemic wave (Table 2), most clinicians noted that access resources significantly diminished between the pre-pandemic phase (84.7%) and the post-pandemic phase (55.9%) [χ^2^(1) = 14.75, *p* < 0.001], as shown in Table 3.

### 3.2. NYS Organizational Facilitators in All Practice Types and All Locations in the Post-Pandemic Period

Current reports [20] suggest that young and older Latinos in urban areas are most at risk for mental health problems and for opiate overdose. In Hypotheses 4 and 5, our questions concerned the pattern of organizational facilitation in the less urbanized parts of the state where Latinos are likely to be at a lower risk, and in types of practice other than SUD practice. Are Latinos outside of the urban sprawl in NYC at a lower risk for SUD and mental health problems (as suggested by national reports) because of better organizational facilitation? What follows are sociodemographic descriptions and organizational facilitator resource descriptions from practitioners across the state in SUD and non-SUD practices. Comparisons of organizational facilitation by two types of location (Downstate and Upstate) and two types of practice (SUD and non-SUD) are offered in the following paragraphs.

#### 3.2.1. Sociodemographic Characteristics for All Post-Pandemic Participants

As shown in Table 4, 150 sampled clinicians throughout Upstate and Downstate New York State who offered SUD and non-SUD treatment ranged in age from 25 to 79 years, with an average age of 49.8 years (*SD* = 12.2). Most participants identified as female (86.9%) and non-Hispanic Caucasian (65.0%), and had earned a graduate degree (92.1%) and had not earned the Credentialed Alcoholism and Substance Abuse Counselor (CASAC) certification (75.3%). Most practiced in a private or outpatient practice setting (48.0%). The proportion of services delivered to Latinos averaged 18.5% as reported by those participants who delivered direct service.

#### 3.2.2. Cultural Facilitator Characteristics for All NYS Locations in the Post-Pandemic Period

To test Hypotheses 4 and 5 concerning the current organizational facilitation of Latino help-seeking for SUD and non-SUD problems throughout the state, the measures for cultural resources (P3) (Table 5) and access resources (P3) were expanded, as described in the Measures section. Access resources are not illustrated in tabular form because there were no significant differences by location for any of the items (location, easy access, flexible hours, and immediate treatment) which were added to the original four items in the post-pandemic survey.

As there were 25 analyses for Table 5, the α was again adjusted using the Šidák correction (α_Šidák_ = 0.0021). There were eight χ^2^ analyses in which the expected cell count fell below 5, prompting the Fisher Exact Test (noted in table). A significantly greater number of participants in Downstate locations reported the presence of the following items in the Cultural Variable list, i.e., intake done by a Latino person [χ^2^ = 14.90, *p* < 0.001] and cultural matching of Spanish-speaking clients with Spanish-speaking counselors [χ^2^ = 10.52, *p* < 0.001]. Note: The four closed-ended questions were retained from the original cultural measure, including the question concerning having no cultural resources—the selection “none of the above”.

#### 3.2.3. Pairwise Comparison of Reports of Post-Pandemic Resources by Downstate and Upstate Locations

Using these expanded measures of cultural resources and access resources, we then reduced each measure to a dichotomous lens for the examination of resources throughout the state in the four domains of interest. As shown in Table 6, the expanded measures revealed that many participants did not have cultural or access resources in the locations in which they practiced. Most importantly, as shown in Table 6, the analysis demonstrated that participants’ experience of organizational facilitation of Latino help-seeking throughout the state did not differ significantly by location in the post-pandemic period. The comparisons of Downstate to Upstate cultural (*p* = 0.06) and access (*p* = 0.27) resources fall short of significance. This analysis rejected Hypothesis 4, that clinicians would report a difference in organizational resources by location. This suggests that the clinician-reported level of organizational resources supporting Latino mental health and substance use disorder help-seeking is consistently low throughout New York State.

#### 3.2.4. Pairwise Comparison of Reports of Post-Pandemic Resources by SUD and Non-SUD Service Types

The types of treatment settings that surveyed participants endorsed included outpatient substance use disorder settings, inpatient or residential settings, and “other”. Participants who selected “other” in the answer set listed mental health, school or university, medical or hospital, community settings, institutional settings like prisons, case management, and administration as types of settings or practices in which they worked. None of the expected cell counts were <5, so the χ^2^ test was used in comparisons. As there were four analyses for Table 7, the α was again adjusted using the Šidák correction (α_Šidák_ = 0.013). In this regard, none of the four χ^2^ analyses were statistically significant (*p* < 0.04).

This analysis rejected Hypothesis 5, that organizational facilitators for Latino help-seeking in NYS would differ significantly by type of help sought. Here, we see that clinicians reported no significant differences in organizational resources facilitating Latino help-seeking, no matter whether the type of help sought was for mental health or substance use disorder problems.

### 3.3. Service Utilization by Latinos

Our previous research [34] demonstrated that the caseload percentage of Latinos in treatment had diminished significantly by 36.9% from 23.8% in the pre-pandemic period to 15% during the pandemic period. In the post-pandemic period, our current research shows that the hourly percentage of services used by Latinos, as reported by Downstate SUD providers, is 18.9%. The reported hourly percentage of statewide SUD and non-SUD services, as reported by respondents, is 18.5%.

## 4. Discussion

Prior to COVID-19, NYS SUD organizations demonstrated greater health delivery cultural competence. Organizational enabling resources included the accommodation of Spanish language needs, cultural facilitators, provision of information about neighborhood, immigration, legal, and insurance status resources, and access facilitation for Latino clinic attendees. Efforts to match of Latino clients with culturally similar staff were routine in the Downstate regions.

### 4.1. Continued Reduction in Organizational Facilitators for Latino Help-Seeking

Study outcomes suggest that a reduction in SUD organizational facilitators for Latino help-seeking in Downstate NYS occurred during the pandemic period (P2), confirming Hypothesis 1a, 1b, and 1c concerning language facilitators, legal and insurance facilitators and cultural facilitators. Only Hypothesis 1d, for access facilitators, was not confirmed because of the rise in telehealth resources during the pandemic period. Organizational facilitators continued eroding or made only negligible recovery in the post-pandemic period, confirming Hypothesis 2a–d and Hypothesis 3a–d. The overall picture is one of significant deterioration. The rejection of Hypotheses 4 and 5 suggests that the organizational deterioration was happening throughout the state and in all types of service organizations, which implies that whatever factors are responsible for the erosion of resources in Downstate SUD organizations are strongly operative in all types or organizations in which licensed clinicians are employed throughout the state.

The findings concerning service utilization suggest that, among Latino clients, the decline in service utilization coincided with reductions in organizational facilitation. Although the post-pandemic estimate of Latino clientele treatment usage in the Downstate area (18.9% of total treatment hours) is not directly comparable to the pre-pandemic Latino caseload proportion (23.8%) or to the significant reduction to 15.0% caseload proportion during the pandemic period, it does point to a continued pattern of lower utilization. Service utilization findings partially support Hypothesis 3, indicating that services for Latinos have not returned to pre-pandemic levels despite some post-pandemic improvement. This interpretation is reinforced by the stability of treatment modality across periods, indicating that changes in service intensity are unlikely to explain the observed differences. Note that the inclusion of mental health services in the post-pandemic period diminishes the estimated statewide Latino service utilization to 18.5%.

The overall picture is one of erosion of enabling organizational SUD resources for Latinos in the Downstate NY area, where most Latinos reside. In a very important way, this coincides with the rise in mental health needs and opiate deaths for both young and older Latinos.

### 4.2. Qualitative Comments

The qualitative data from the October–November contest, generated by 168 respondents, used open-ended questions to ask service providers how services for Latinos could be improved. Answers were organized into categories or themes by one author. This effort was made to understand the most current clinician thoughts about the improvement of services. Several themes emerged in the responses of the respondents. A central theme was the critical importance of organizational cultural responsiveness or cultural competence. When clients and providers engage across cultural boundaries, the interaction is shaped by existing structural and relational power differentials. In such contexts, the client—particularly when positioned outside the dominant culture—may be placed at a distinct disadvantage. Recognizing and actively addressing these imbalances is essential to ethical and effective practice. Most respondents noted this theme and had ideas about how to impact the problem, e.g., [programs would be improved by] “staff gaining more education on the Latino culture and differences in each Latino native country’s culture and dialect”, [programs would be improved by] “cultural humility, don’t assume you know anything about the culture, be curious and engage, also having bilingual providers who are from a similar culture…”, and [programs would be improved by] “acknowledgement of differences between cultural groups and level of acculturation (New immigrants vs. second or third generation citizens)”. One provider mentioned how one organizational approach to cultural responsiveness was to do cultural matching, i.e., matching incoming Latino clients with Latino staff. This is also mentioned in some literature as a strategy that increases engagement [14]. In the first wave of the study, the client caseloads of Latinos were significantly higher for Latino staff than for non-Latino staff, demonstrating the results of this organizational matching effort. However, the proportional hours of Latino service utilization and the ethnicity of the staff members in the P3 period demonstrated no significant relationship. This is another indication of diminished organizational cultural responsiveness.

Most indicated that the language of service delivery was important, and that cultural nuances may improve the alliance between counselor and client. Latino communities of the New York region are marked by a great diversity in language use in both English and Spanish. One mental health practitioner linked the offering of services in the language of the client to more likelihood of the use of services, i.e., [service in the language of the client] “provides the opportunity for greater clarity and supportive access for Latinos who often don’t seek mental health services. To make mental health more accessible [through language] may increase the chances of services being actually used”. Another clinician suggested “incorporating symbolism relevant to the Latina person is crucial [for mental health treatment]. That includes regional colloquialisms of the language”.

Another important theme centered on the safety of those in treatment. Many respondents were aware of the difficulties that immigrants face because of the conflict between receiving treatment and having the treatment facility be required to cooperate with authorities from Immigration and Customs Enforcement. Respondents reported that the conflicting organizational roles had elicited fears of imprisonment and deportation in undocumented Latino clientele. One practitioner said, “Safety is now an issue, and Latinos consider mental health treatment unsafe”. Another felt the issue broadened into all services, “I believe the DEI ICE travesty makes many refugees have their children stay away from school and adults from work”.

The implementation of programmatic safety through strict confidentiality and telehealth was emphasized by respondents, e.g., “Privacy and confidentiality must be absolutely guaranteed, and communicated clearly. That is a basic tenet for all clients, but even more so right now for Latino clients” and “Bilingual telehealth services are important for those that fear ICE and are afraid to travel, even if they are documented”.

Of those who suggested improvements for treatment, many voiced the idea that the stress for undocumented Latino immigrant clientele should be understood by clinicians as traumatic, e.g., [Latinos are a] “high risk population—we need to provide trauma informed culturally competent care”. Other treatment suggestions were that a strengths-based or an empowerment approach should be implemented in the work with these individuals, e.g., [programmatic improvements would be] “strengths based programs, validating trauma response to discrimination” and “programs related to trauma-based discrimination due to current immigration policy in the US, i.e., finding strength when facing discrimination…”.

### 4.3. Study Limitations

The outcomes of this study have limited generalizability due to several limitations of the study method. The results should be viewed through a lens that acknowledges nonresponse error, potential selection bias, historical biases, and unit of analysis issues.

#### 4.3.1. Nonresponse Error

The response rate in a study with a survey as a collection instrument is important because a low response rate is more likely to yield a sample of a population which is not representative of the characteristics of the entire population. This study had 357 respondents out of 43,000 potential respondents, which is a low (<1%) response rate. The low response rate calls into question the representativeness of the sample. This alone does not bias the results in any particular direction but may be part of the reason that study demographics were very different over the three waves.

#### 4.3.2. Potential Selection Bias

Because of the convenience sampling method, there is a possibility that providers who were interested in health equity for Latinos may have self-selected into the sample. Professional perception of organizational resources may have been affected by bias. This may have skewed outcomes in the positive or in the negative direction.

#### 4.3.3. Effects of History

Sampling over time cannot control or account for the impact of influences which are not included in the study and may affect outcomes. The longer the time period over which the study occurs, the more likely that outcomes will be affected by history. The pandemic period certainly was related to a reduction in Latino clientele in treatment [34,35]. However, other factors may have affected the reduction in the clinician report of organizational resources which are not explained by the study.

The significant changes in provider ethnicity, education and certification observed across the waves of the study, the substantially fewer respondents working in substance use treatment in the P2 period, and the increase in Spanish-speaking clinicians and translators during the overall decline of language resources are not explained by the study. These shifts may be due to factors the study overlooked and may have resulted in biased outcomes.

#### 4.3.4. Unit of Analysis

Using the individual provider as a unit of analysis and reporting organizational outcomes creates a measurement issue. Provider reports can only suggest how organizations are being impacted by external events and how they are responding. Using the organization as a unit of analysis would require a different sampling technique than the one we used for this study. Also, the post-pandemic measure of service utilization reflects service intensity (hours) rather than client representation (caseload percentage) and so it cannot be directly compared to earlier figures. Generally, the outcomes of the study may point to trends but cannot be regarded as representative.

#### 4.3.5. Measure of Cultural Competence

Limitations also include the measure of agency cultural competence, which was not checked with a cultural advisor, and has been superseded in the public domain.

Despite these limitations, the outcomes of the study provide important information about how New York State service providers gauge resources which facilitate the help-seeking efforts of Latinos in the organizations in which they worked over the period from 2018 to 2024. The study also allowed clinicians to suggest how New York State organizations can improve the facilitation of Latino help-seeking.

## 5. Conclusions

The outcomes of this study suggest that the disparity that exists in NYS healthcare delivery deepened during the pandemic, and that there has been little or no recovery in organizational resources that facilitate Latino access. Previously, our research had suggested that barriers to treatment were too steep for Latinos to obtain assistance through traditional SUD treatment avenues during the pandemic [34]. Although treatment access for many improved with the rise in telehealth, the increase was not sufficient to counter the overall drop in access resources. Our current research suggests that the organizational facilitators for Latino help-seeking throughout the state, as reported by NYS clinicians, are still sagging. Our inference is that the organizational bulwark against the rise in opiate use and mental health problems which are already affecting the youngest and oldest Latinos in the urban area of the state is insufficient to mitigate a growing post-pandemic public health risk for younger and older Latinos.

### Implications for Health Equity

In the past, New York State, a US state with a relatively high density of Latinos compared to most other US states, maintained a better health insurance and institutional safety net for this vulnerable population [36]. The outcomes of this study suggest that NYS organizational resources are dwindling, resulting in reduced access to and lower quality of mental health and substance use disorder care for Latinos. Since mental health problems and drug use are increasing for younger and older Latinos in urban areas [20], and the current sociopolitical climate is unsupportive to health and safety issues [37], inferences from public health models such as the Vulnerability Model predict poorer health outcomes for Latinos in this post-pandemic period.

The use of the Vulnerability Model to craft interventions suggests intervening to reduce health inequity at the policy, community, organizational and individual levels. Primary and targeted prevention strategies, low-cost cornerstones of public health practice, can be designed and implemented at all those levels.

Inferences from the results of the current National Survey on Drug Use and Health (NSDUH) [20] emphasize the importance of targeted outreach and resource linkage for younger and older Latino populations in this post-pandemic period. Prevention efforts targeting the Latino community should prioritize geographic areas with higher Latino population density, including Downstate New York and increasingly urbanized regions of Upstate New York, where Latino communities are expanding. Organizational strategies should incorporate facilitators in the four domains mentioned in this study: Spanish language accommodation, legal and insurance navigation, cultural responsiveness, and physical access. Organizational prevention efforts should also integrate clinicians’ field-based expertise, emphasizing programmatic initiatives that enhance client safety and strengthen organizational cultural competence.

## Figures and Tables

**Table 1:** Sociodemographic characteristics for downstate study participants in SUD practice by wave (*N* = 241).

Variables	Pre-Pandemic (*n* = 176)		Pandemic (*n* = 31)		Post-Pandemic (*n* = 34)		Total (*N* = 241)		*p*
	*M*	*SD*	*M*	*SD*	*M*	*SD*	*M* (*SD*)	Range	
Age	53.5	10.6	52.6	13.1	49.5	11.3	52.8 (11.1)	25–76	0.17
Caseload % Latinos	23.8	25.0	15.0	35.2				0–100	0.02
% SvcHrs Delivered to Latinos					18.9	27.4		0–100	
	*n*	%	*n*	%	*n*	%	*n*	%	
Gender									0.51
Female	118	67.1	19	61.3	24	70.6	161	66.8	
Male	56	31.8	12	38.7	8	23.5	76	31.5	
Other	0	0.0	0.0	0.0	1	2.9	1	0.4	
Race/Ethnicity									<0.001
Black	51	29.0	3	9.7	3	8.8	57	23.7	
Hispanic	34	19.3	5	16.1	2	5.9	41	17.0	
White	66	37.5	20	64.5	18	52.9	104	43.2	
Other	17	9.7	3	9.7	10	29.4	30	12.5	
Education									0.002
HSD or Associate’s	47	26.7	9	29.0	2	5.9	58	24.1	
Bachelor’s	43	24.4	5	16.1	3	8.8	51	21.2	
Graduate Degree	79	44.9	17	54.8	28	82.4	124	51.5	
CASAC									<0.001
Yes	166	94.3	29	93.6	16	47.1	211	87.6	
No	9	5.1	1	3.2	18	52.9	28	11.6	
Practice Type									0.13
Private/OP	74	42.1	11	35.5	22	64.7	107	44.4	
IOP/IP/Res	55	31.3	10	32.3	7	20.6	72	29.9	
Other	46	26.1	10	32.3	5	14.7	61	25.3	

Note. HSD = high school diploma. OP = outpatient. IOP = intensive outpatient. IP = inpatient. Res = residential. *p*-values reflect tests of mean differences for age (analysis of variance) and Tests of Association (Pearson χ^2^) for categorical variables.

**Table 2:** Descriptive statistics (frequencies and percentages) for resource variables (*N* = 241).

Resource Variables	Pre-Pandemic (*n* = 176)		Pandemic (*n* = 31)		Post-Pandemic (*n* = 34)		Total (*N* = 241)		*p*
	*n*	%	*n*	%	*n*	%	*n*	%	
Language									
Translators	32	18.2	9	29.0	16	47.1	57	23.2	
Spanish-speaking clinicians	68	38.6	16	51.6	20	58.8	104	43.2	
Spanish signage/brochures	29	16.5	9	29.0	9	26.5	47	19.5	
No resources	17	9.7	11	35.5	10	29.4	38	15.8	<0.001
Legal/Financial									
Telephone legal resources	40	22.7	8	25.8	13	38.2	61	25.3	
Papers/Brochures on rights	29	16.5	7	22.6	10	29.4	46	19.1	
Instructions to access services	44	25.0	7	22.6	10	29.4	61	25.3	
No resources	24	13.6	16	51.6	18	52.9	58	24.1	<0.001
Cultural *									
Childcare provided	16	9.1	1	3.3	4	11.8	21	8.7	
Phone numbers provided	40	22.7	12	38.7	7	20.6	59	24.5	
Outreach provided	27	15.3	5	16.1	3	8.8	35	14.5	
None of the above	31	17.6	14	45.2	26	76.5	71	29.5	<0.001
Access									
Telehealth	14	8.0	8	25.8	13	38.2	35	14.5	
Late hours	43	24.4	10	32.3	11	32.4	64	26.6	
Transportation	27	15.3	8	25.8	8	23.5	43	17.8	
None of the above	27	15.3	10	32.3	15	44.1	52	21.6	<0.001

Note. As participants were encouraged to select as many response options as applicable to their practice, the frequencies and percentages do not equal the sample size. The *p*-values reflect the χ^2^ Test of Association to determine whether there were differences in the frequencies of the “No resources”/”None of the above” options by wave. * Childcare is cultural when the language of the childcare environment is Spanish or bilingual. Outreach is cultural when it, for example, is adjusted to reach out to locations where Latinos usually frequent, or if it is done in Spanish or is bilingual.

**Table 3:** Pairwise comparisons for frequencies and percentages for resource variables by wave (*N* = 241).

Resource Variables	Pre-Pandemic (P1) (*n* = 176)		Pandemic (P2) (*n* = 31)		Post-Pandemic (P3) (*n* = 34)		*p*
	*n*	%	*n*	%	*n*	%	
Language							
At least one resource	159	90.3	20	64.5	24	70.6	
No resources	17 ^a^	9.7	11 ^a^	35.5	10	29.4	<0.001
Legal/Financial							
At least one resource	152	86.4	15	48.4	16	47.1	
No resources	24 ^ab^	13.6	16 ^a^	51.6	18 ^b^	52.9	<0.001
Cultural							
At least one resource	145	82.4	17	54.8	8	23.5	
None of the above	31 ^ac^	17.6	14 ^ab^	45.2	26 ^bc^	76.5	<0.001
Access							
At least one resource	149	84.7	21	67.7	19	55.9	
None of the above	27 ^a^	15.3	10	32.3	15 ^a^	44.1	<0.001

Note. The *p*-values reflect the χ^2^ Test of Association to determine whether there were differences in the frequencies of the “No resources”/”None of the above” and “At least one resource” options by wave. As there were three pairwise comparisons for each resource variable (P1–P2, P2–P3, P1–P3), the α_Šidák_ = 0.017. Row cells for each resource category (Language, Legal/Financial, Cultural, Access) with the same superscript letters are significantly different from one another (α_Šidák_ < 0.017).

**Table 4:** Sociodemographic characteristics of all clinicians in the post-pandemic wave (*N* = 150).

Variables	*n*	%	*M* (*SD*)	Range
Age	140		49.8 (12.2)	25–79
% SvcHrsDelivered to Latinos	92		18.5 (28.8)	0–100
Gender	137			
Female	119	86.9		
Male	18	13.1		
Education	140			
HSD or Assoc.	4	2.9		
Bachelors	7	5.0		
Graduate Degree	129	92.1		
Ethnicity	140			
Black	14	10.0		
Hispanic	9	6.4		
White	91	65.0		
Other	26	18.6		
Clin. Licensure	150			
Licensed	113	75.3		
Certified	37	24.7		
Offering SUD tx	150			
No	98	65.3		
yes	52	34.7		
Type of Practice	150			
Private/OP	72	48.0		
IOP/IP/Res	17	11.3		
Other	61	40.7		

Note. HSD = high school diploma. OP = outpatient. IOP = intensive outpatient. IP = inpatient. Res = residential.

**Table 5:** Cultural items included in cultural resources measure for the post-pandemic wave (*N* = 144).

Cultural Variables	Downstate (*n* = 95)		Upstate (*n* = 49)		Total (*n* = 144)		χ^2^	*p*
	*n*	%	*n*	%	*n*	%		
Cultural intake by Hispanic	34	35.8	3	6.1	37	25.7	14.90	<0.001
Cultural matching	31	32.6	4	8.2	35	24.3	10.52	<0.001
Cultural environment	12	12.6	4	8.2	16	11.1	0.65	0.42
Cultural relevant assessment	27	28.4	9	18.4	36	25.0	1.74	0.16
Cultural appropriate framework	24	25.3	8	16.3	32	22.2	1.49	0.22
Cultural intervention model	15	15.8	5	10.2	20	13.9	0.84	0.34
Cultural prevention	24	25.3	8	16.3	32	22.2	1.49	0.22
Cultural brief services	19	20.0	11	22.4	30	20.8	0.12	0.73
Cultural community services	20	21.1	7	14.3	27	18.8	0.97	0.32
Cultural couples services	10	10.5	2	4.1	12	8.3	-	0.22
Cultural crisis services	16	16.8	9	18.4	25	17.4	0.05	0.82
Cultural education services	20	21.1	3	6.1	23	16.0	5.37	0.02
Cultural individual services	25	26.3	14	28.6	39	27.1	0.08	0.77
Cultural natural helpers	8	8.4	5	10.2	13	9.0	-	0.76
Cultural outreach	16	16.8	5	10.2	21	14.6	1.14	0.29
Cultural resource linkage	23	24.2	11	22.4	34	23.6	0.06	0.81
Cultural Latino operation	4	4.2	0	0.0	4	2.8	-	0.30
Cultural facilities used	6	6.3	3	6.1	9	6.3	-	0.99
Cultural ties provided	12	12.6	4	8.2	16	11.1	0.65	0.42
Latino advocate	5	5.3	2	4.1	7	4.9	-	0.99
Cultural advisor use	5	5.3	1	2.0	6	4.2	-	0.66
Cultural evaluator provided	6	6.3	1	2.0	7	4.9	-	0.42
Cultural childcare provided	9	9.5	2	4.1	11	7.6	-	0.44
Cultural Ph. numbers provided	10	10.5	5	10.2	15	10.4	0.00	0.96
None of the above	41	43.2	30	61.2	71	49.3	3.63	0.06

Note. All analyses utilized the χ^2^ test except where the χ^2^ value is reported as “-”. In these cases, there was a violation of the expected cell count assumption in χ^2^. For that reason, the *p*-value reflects the result from the Fisher Exact Test.

**Table 6:** Resource variables by location (Downstate vs. Upstate) in the post-pandemic wave (*N* = 144).

Resource Variables	Downstate (*n* = 95)		Upstate (*n* = 49)		χ^2^(1)	*p*
	*n*	%	*n*	%		
Language					2.49	0.12
At least one resource	65	68.4	27	55.1		
No resources	30	31.6	22	44.9		
Legal/Financial					0.03	0.87
At least one resource	46	48.4	23	46.9		
No resources	49	51.6	26	53.1		
Cultural					3.63	0.06
At least one resource	54	56.8	19	38.8		
None of the above	41	43.2	30	61.2		
Access					1.21	0.27
At least one resource	42	44.2	17	34.7		
None of the above	53	55.8	32	65.3		

**Table 7:** Resource variables by provision of substance use treatment (Yes vs. No) in the post-pandemic wave (*N* = 144).

Resource Variables	No (*n* = 92)		Yes (*n* = 52)		χ^2^(1)	*p*
	*n*	%	*n*	%		
Language					0.08	0.78
At least one resource	58	63.0	34	65.4		
No resources	34	37.0	18	34.6		
Legal/Financial					0.14	0.71
At least one resource	43	46.7	26	50.0		
No resources	49	53.3	26	50.0		
Cultural					4.07 *	0.04
At least one resource	11	12.0	13	25.0		
None of the above	81	88.0	39	75.0		
Access					4.04 *	0.05
At least one resource	32	34.8	27	51.9		
None of the above	60	65.2	25	48.1		

* *p* < 0.05.

## Data Availability

The datasets generated by this study are available by contacting the corresponding author in writing with a request. The data are not shared in a public repository for ethical reasons.

## Supplementary Materials

The following supporting information can be downloaded at: https://www.mdpi.com/article/10.3390/ijerph23050628/s1, Table S1: List of NY counties included in the analysis of Downstate and Upstate NY; File S1: Provider survey; File S2: Provider survey in Spanish; File S3: Provider survey for qualitative comments.
