# Supplementary material for: Left Out and At Risk: Post-Pandemic Continuation of Organizational Service Reduction in Metropolitan New York City Coincides with Rise in Opiate Use and Mental Health Problems for Latinos
Source: Int J Environ Res Public Health. 2026 May 8;23(5):628. doi: 10.3390/ijerph23050628 (PMC13206180; doi:10.3390/ijerph23050628)
Supplement: Supplementary file 1 [file ijerph-23-00628-s001.zip › Table S1 List of NY counties included in the analysis of Downstate and Upstate NY.pdf]

Table S1. List of NY counties included in the analysis of Downstate and Upstate NY.

County - Downstate	County - Upstate	Density Latinos 2000 (%)	Density Latinos 2020	2022
		>5 = yellow	>10	
	Albany	3<5	6.5	
	Allegany	1<5	<5	
Bronx		48	54.8	806463
	Broome	2<5	5	
	Cattaraugus	1<5	<5	
	Cayuga	2<5	<5	
	Chautauqua	4<5	11-12	
	Chemung	2<5	<5	
	Chenango	1<5	<5	
	Clinton	2<5	<5	
	Columbia	3<5	<5	
	Cortland	1<5	<5	
	Delaware	2<5	<5	
Dutchess		6	14-16	**43,265 in 2022**
	Erie	3<5	6.3	
	Essex	2<5	<5	
	Franklin	4<5	<5	
	Fulton	2<5	<5	
	Genessee	1<5	<5	
	Greene	4<5	7	
	Hamilton	1<5	<5	
	Herkimer	1<5	<5	
	Jefferson	4<5	8	
Kings		20	18.9	516426
	Lewis	1<5	<5	
	Livingston	2<5	<5	
	Madison	1<5	<5	
	Monroe	5	9.6	
	Montgomery	7	15.2	
Nassau		10	18.4	256425
New York County		27	23.8	402,640

	Niagara	1<5	<5	
	Oneida	3<5	5.8	
	Onondaga	2<5	5.7	
	Ontario	2<5	5.6	
Orange		12	22	93,600
	Orleans	4<5	5.4	
	Oswego	1<5	<5	
	Otsego	2<5	4.3 <5	
Putnam		6	20.8	**18,713 2022**
Queens		25	27.8	667,861
	Renssalaer	2<5	10.3	
Richmond		12	19.6	96,960
Rockland		10	19.5	66,451 (census)
	St. Lawrence	2<5	<5	
	Saratoga	1<5	<5	
	Schenectady	3<5	7.7	
	Schoharie	2<5	<5	
	Schuyler	1<5	<5	
	Seneca	2<5	<5	
	Steuben	1<5	<5	
Suffolk		11	21.8	332,959
Sullivan		9	20	14,957 2022est
	Tioga	1<5	<5	
	Tomkins	3<5	7	
Ulster		6	12	**21,070 in 2022**
	Warren	1<5	<5	
	Washington	2<5	<5	
	Wayne	2<5	<5	
Westchester		16	26.8	269,334
	Wyoming	3<5	<5	
	Yates	1<5	<5	
