# Supplementary material for: Left Out and At Risk: Post-Pandemic Continuation of Organizational Service Reduction in Metropolitan New York City Coincides with Rise in Opiate Use and Mental Health Problems for Latinos
Source: Int J Environ Res Public Health. 2026 May 8;23(5):628. doi: 10.3390/ijerph23050628 (PMC13206180; doi:10.3390/ijerph23050628)
Supplement: Supplementary file 1 [file ijerph-23-00628-s001.zip › File S1 Provider survey.pdf]

2023-6_W_CBTpre_en

Welcome: This is the Essential Cognitive Behavior Therapy for Substance Use Disorders course Pretest

IF the print is too small, please use Control and the "+" key to make it larger and easier to read. If the print is too large, please use Control and the "-" key to make it smaller. **Thank you for taking the time to answer these questions concerning your practice. We're trying to see how services can be improved for Latinos and to counteract the toll that the opioid overdose epidemic has taken on services. The de-identified answers will be used in current and future research supported by Connections Counseling PLLC. Any personal answers you give will be kept confidential to the full extent of the law. The research questions have been approved by the North Star IRB, reachable at info@northstarreviewboard.org. No other organization has participated in designing nor has any other organization approved this research effort. You are not obligated to complete this pretest; however, you will not be able to enter the course if you do not complete it. The amount of time to complete this questionnaire is usually about 15 minutes. Your feedback is very important in order to help us improve clinical services.**

* 1. Please enter the 6-character code that was sent to you to log into this course.

* 2. Please enter your email address so we can match your pretest and posttest answers.

* 3. Please confirm your email address below.

2023-6_W_CBTpre_en

Essential Cognitive Behavior Therapy for Substance Use Disorders pretest Pt 1

In the questions below, "Hispanic / Latino" is defined in the same way that the US Census Bureau defines Hispanic or Latino, that is, "a person of Cuban, Mexican, Puerto Rican, South American, or Central American or other Spanish culture or origin, regardless of race."

* 4. Have you had previous clinical training in the use of Cognitive Behavioral Therapy methods with clients with Substance Use Disorders? Note: 'clinical,' in this case, means the nuts nad bolts of how to use the technique - for example, experiential training would qualify as 'clinical'.

- ◯ Yes
- ◯ No

* 5. 74% of clinicians work with or have worked with clients with SUDs (substance use disorders). How many years have you worked with clients with SUDs? (Please put a whole number in the box below. If you are an intern practicing with SUD clients, please put 1 in the box.)

[ ]

* 6. At the present time, are you offering any in-person client assessment or treatment for any Substance Use Disorder? (This may include individual, group or family treatment. This may include discussion of relapse prevention strategies or instruction in Substance Use Disorders.)

- ◯ Yes
- ◯ No

* 7. What type of clinical licensure/ certification do you have? (choose all that apply)

- ☐ Social work licensure
- ☐ CASAC
- ☐ Mental Health Counseling
- ☐ Marriage and Family Counseling
- ☐ I do not have a license
- ☐ Other (please specify)

[ ]

* 8. In what country are you licensed to practice? (If you are licensed in more than one country, please choose the one in which you practice most of the time.)

- ◯ I am not licensed to practice
- ◯ United States
- ◯ Canada

* 9. In what state/province are you licensed to practice? (If you are licensed in more than one state/province, please choose the one in which you practice most of the time.) (Scroll down to find Canadian provinces)

[ ]

* 10. If you practice in New York State, in what NY county do you practice? (If more than one, choose the one in which you practice most of the time. If you no longer practice in NY, please choose the first response - "I do not or no longer practice in New York State.")

* 11. Practice Type (Which one most accurately describes your work setting?)

- Private or Outpatient Practice
- Intensive Outpatient/ Inpatient or Residential Practice
- Other (please specify)

* 12. Did you see clients in the past two weeks (for intake, assessment, in groups/families or individually)?

- Yes
- No

* 13. In the past two weeks, how many hours did you spend providing clinical services to your clients? (If you have no clients, please put NA in the box below. Otherwise, please enter a number.)

* 14. In the past two weeks, how many hours did you spend providing clinical services to Latino clients? (If you have no clients, please put NA in the box below. Otherwise, please enter a number.)

2023-6_W_CBTpre_en

Treatment Facilitation for Latino clients Part 2

Many things discourage Latinos from getting help for mental health or substance use disorders. The questions in this survey concern barriers/facilitators for Latino clients who are looking for help with those disorders. Please answer the questions as best you can.

* 15. LANGUAGE: The resources listed below might help Latinos overcome language barriers in order to get help. Please check the ones that your organization (including your private practice, if any) offers. If you know of other ways that your clinic helps Latinos overcome language barriers, please list them in the space marked "comments."

- [ ] Translator for Spanish speakers
- [ ] Clinicians who speak Spanish
- [ ] Signage or brochures in Spanish
- [ ] Telehealth in Spanish for virtual treatment
- [ ] None of the above

Comment : If there are other ways in which your organization facilitates the Latino use of services through language resources, please explain below

* 16. LEGAL & FINANCIAL: The resources listed below might help Latinos overcome legal or financial barriers in order to get help. Please check the ones that your organization (including your private practice, if any) offers. If you know of other ways that your clinic helps Latinos overcome legal or financial barriers, please list them in the space marked "comments."

- [ ] A list of phone numbers for legal help in Spanish
- [ ] A paper or brochure in Spanish concerning rights of undocumented Latinos
- [ ] Instructions In Spanish to help Latino clients get access to coverage for services
- [ ] None of the above

Comment : If there are other ways in which your organization facilitates the Latino use of services through legal or financial resources, please explain below

* 17. CULTURAL: The resources listed below might help Latinos overcome cultural barriers in order to get help. Please check the ones that your organization (including your private practice, if any) offers. If you know of other ways that your clinic helps Latinos overcome cultural barriers, please list them in the space marked "comments."

- [ ] Intake done by a Latino/Hispanic person
- [ ] Matching of Latino clients with Latino staff
- [ ] An environment that reflects the Latino cultures
- [ ] A culture-relevant assessment
- [ ] Staff frames problems in a cultural context
- [ ] Staff uses a cultural-specific intervention model
- [ ] Culturally-informed prevention services
- [ ] Culturally-informed brief services
- [ ] Spanish-speaking child services at the clinic for Latino children for parents who are attending treatment
- [ ] Culturally-informed community services
- [ ] Culturally-informed couple services
- [ ] Culturally-informed crisis services
- [ ] Culturally-informed Education services
- [ ] Culturally-informed individual services
- [ ] The use of natural helpers and/or a systems focus
- [ ] Culturally-informed Outreach services
- [ ] Resource linkage services
- [ ] The organization is operated by the Latino community
- [ ] The organization uses existing minority community facilities
- [ ] The organization has ties to the minority community
- [ ] The organization uses an advocate for services from the Latino community
- [ ] The organization uses a person(s) from the Latino community as an adviser
- [ ] The organization uses a person(s) from the Latino community as an evaluator
- [ ] A list of phone numbers for Neighborhood resources to welcome Latinos, such as a Community Outreach Team
- [ ] Organizational Outreach to local Churches that Latinos may attend.
- [ ] None of the above
- [ ] Comment : If there are other ways in which your organization facilitates the Latino use of services through culturally sensitive resources, please explain below

* 18. ACCESS: The resources listed below might help Latinos overcome access barriers in order to get help. Please check the ones that your organization (including your private practice, if any) offers. If you know of other ways that your clinic helps Latinos overcome access barriers, please list them in the space marked "comments."

- [ ] Telehealth resources in Spanish for those Latinos who want treatment and can't come to the clinic because of barriers
- [ ] Late clinic hours for those who are working full time jobs and also trying to get treatment.
- [ ] Transportation provided for those who do not have transportation to get to the clinic.
- [ ] Location of the organization in a Latino community
- [ ] Easy access for Latinos (on bus or subway routes, or easy parking availability)
- [ ] Flexible hours/appointments/ home visits
- [ ] The organization offers immediate (within a day or week) treatment
- [ ] None of the above

Comment : If there are other ways in which your organization facilitates the Latino use of services through access resources, please explain below

* 19. OPIATE-SPECIFIC: The resources listed below might help Latinos overcome access barriers in order to get help specifically for opiate-related SUDs. Please check the ones that your organization (including your private practice, if any) offers. If you know of other ways that your clinic helps Latinos overcome these barriers, please list them in the space marked "comments."

- [ ] Medication Assisted Treatment
- [ ] On-site physician who can prescribe suboxone
- [ ] Mindfulness-Oriented Recovery Enhancement for opioid misuse (per EL Garland)
- [ ] Referral for concurrent treatment for opiate use disorder
- [ ] None of the above

Comment : If there are other ways in which your organization facilitates the Latino use of services through opiate-specific resources, please explain below

* 20. OTHER: How else does your program (including your private practice, if any) facilitate the Hispanic/Latino clients and/or Spanish-speaking clients to access services? Please explain in a few sentences in the space below.

CHARACTERISTICS of your PRACTICE Part 3

Please provide us with this important information about you and your practice. Please answer to the best of your ability

* 21. What is your gender?

- ◯ Female
- ◯ Male
- ◯ Other

* 22. What is your age? Please enter a whole number (not a word, but rather a digit) in the box below.

[ ]

* 23. With which ethnicity do you identify?

- ◯ Black/African American
- ◯ Hispanic/Latino/Spanish descent
- ◯ Asian or South Asian
- ◯ Native Hawaiian or other Pacific Islander
- ◯ Caucasian not Hispanic/Latino/Spanish descent
- ◯ Native American or Alaskan native
- ◯ Two or more of the above
- ◯ Other

* 24. What is the highest level of education you've completed?

- ◯ High School graduate
- ◯ Associate's Degree
- ◯ Bachelor's Degree
- ◯ Master's Degree
- ◯ Doctoral Degree

2023-6_W_CBTpre_en

Final Thoughts

The questions on this page simply request your assurance that you understand the process of getting your certificate, and request that you possibly provide us with contact information for those who might benefit from this training.

* 25. Please confirm that you understand that one of the requirements of earning your certificate is 'attendance', like any 'live' course. Your electron presence is noted and recorded.

- ◯ Yes
- ◯ No

26. If you know of any other person that would benefit from the free CBT training that is being offered in both English and Spanish, please let them know about it. We would be happy to reach out to them if you would put contact information in the space below.

2023-6_W_CBTpre_en

Congratulations! - here's the next step

Thank you so much for choosing this Essential Cognitive Behavioral Therapy for Substance Use Disorders training! You've completed the pretest. Your information will help us improve services for other SUD clinicians. Please click on the Done button to begin the course. Please contact Ruth Campbell at ruth@connectionscounseling.org with any questions. Or call her at 845-255-5022.
