# Supplementary material for: Left Out and At Risk: Post-Pandemic Continuation of Organizational Service Reduction in Metropolitan New York City Coincides with Rise in Opiate Use and Mental Health Problems for Latinos
Source: Int J Environ Res Public Health. 2026 May 8;23(5):628. doi: 10.3390/ijerph23050628 (PMC13206180; doi:10.3390/ijerph23050628)
Supplement: Supplementary file 1 [file ijerph-23-00628-s001.zip › File S2 Provider survey in Spanish.pdf]

2026-6_W_TCCentrada_es

Bienvenido(a): esta es la prueba de entrada del curso de Terapia Cognitivo-Conductual Esencial

***Si la letra es demasiado pequeña, utilice la tecla Control y "+" para agrandarla y facilitar su lectura. Si la letra es demasiado grande, utilice la tecla Control y "-" para reducirla.* Gracias por tomarse la molestia de responder a estas preguntas sobre su práctica clínica. Estamos tratando de determinar cómo se pueden mejorar los servicios para atender mejor a la población latina y contrarrestar los estragos causados por la epidemia de sobredosis de opioides sobre los servicios. Sus respuestas anonimizadas se utilizarán en investigaciones promovidas por Connections Counseling PLLC. Cualquier respuesta personal que usted proporcione se mantendrá confidencial hasta donde la ley lo permita. Las preguntas de investigación han sido aprobadas por la Junta de Revisión Institucional do North Star. Ninguna otra organización ha participado en el diseño de esta investigación ni ha aprobado esta iniciativa. Usted no tiene la obligación de realizar esta prueba; sin embargo, no podrá ingresar al curso si no lo hace. El tiempo requerido para completar este cuestionario suele ser de unos 15 minutos. Sus comentarios son muy importantes para ayudarnos a mejorar los servicios clínicos.**

* 1. Introduzca el código de 6 caracteres que se le envió para iniciar sesión en el curso.

* 2. Introduzca su dirección de correo electrónico para que podamos emparejar sus respuestas de la prueba de entrada y la prueba de salida.

* 3. Confirme su dirección de correo electrónico a continuación.

2026-6_W_TCCentrada_es

Prueba de entrada: Terapia Cognitivo-Conductual Esencial para Trastornos por Consumo de Sustancias. 1

En las preguntas siguientes, "hispano / latino" se define de la misma manera que la Oficina del Censo de EE. UU. define hispano o latino, es decir, "una persona de cultura u origen cubano, mexicano, puertorriqueño, sudamericano o centroamericano o de otra cultura u origen español, independientemente de su raza".

* 4. ¿Ha recibido capacitación clínica previa en el uso de métodos de terapia cognitivo-conductual con pacientes con trastornos por consumo de sustancias? Nota: “clínica”, en este caso, hace referencia a los principios básicos de cómo utilizar la técnica; por ejemplo, la capacitación experiencial se consideraría "clínica".

- ◯ Si
- ◯ No

* 5. El 74 % de los clínicos trabajan o han trabajado con pacientes con TCS (trastornos por consumo de sustancias). ¿Cuántos años lleva trabajando con pacientes con TCS? (Señale un número entero en la casilla de abajo. Si es un practicante que ejerce con pacientes con TCS, escriba 1 en la casilla).

[ ]

* 6. En la actualidad, ¿ofrece alguna evaluación o tratamiento en persona a los clientes por algún trastorno por consumo de sustancias? (Esto puede incluir tratamiento individual, de grupo o familiar; la discusión de estrategias de prevención de recaídas o formación en trastornos por consumo de sustancias).

- ◯ Sí
- ◯ No

* 7. ¿Qué tipo de licencia clínica tiene? (Elija todas las que correspondan)

- ☐ Licencia de trabajo social
- ☐ CASAC
- ☐ Asesoramiento en salud mental
- ☐ Asesoramiento matrimonial y familiar
- ☐ No tengo licencia
- ☐ Otros (especifique)

[ ]

* 8. ¿En qué país está autorizado(a) a ejercer? (Si es más de uno, elija el país en el que ejerce la mayor parte del tiempo).

- ◯ No estoy autorizado(a) para ejercer
- ◯ Estados Unidos
- ◯ Canadá

* 9. ¿En qué estado/provincia está autorizado(a) a ejercer? (Si es más de uno, elija el estado o provincia en el que ejerce la mayor parte del tiempo). (Desplácese hacia abajo para encontrar las provincias canadienses).

[ ]

* 10. Si ejerce en el estado de Nueva York, ¿en qué condado de Nueva York lo hace? (Si es más de uno, elija aquel en el que ejerza la mayor parte del tiempo. Si ya no ejerce en Nueva York, elija la primera respuesta: "No ejerzo o he dejado de ejercer en el Estado de Nueva York").

[ ]

* 11. Tipo de práctica (¿Qué opción describe mejor su entorno de trabajo?)

- ○ Práctica privada o ambulatoria
- ○ Práctica intensiva ambulatoria/ hospitalaria o residencial
- ○ Otros

Otros (especifique)

[ ]

* 12. ¿Atendió a pacientes en las últimas dos semanas (para admisión, evaluación, en grupos/familias o individualmente)?

- ○ Sí
- ○ No

* 13. Durante las últimas dos semanas, ¿cuántas horas pasó proporcionando servicios clínicos a sus clientes? (Si no tiene clientes, escriba NA en el casillero siguiente. De otro modo, ingrese un número.)

[ ]

* 14. Durante las últimas dos semanas, ¿cuántas horas pasó proporcionando servicios clínicos a clientes de origen latino? (Si no tiene clientes, escriba NA en el casillero siguiente. De otro modo, ingrese un número.)

[ ]

2026-6_W_TCCentrada_es

Facilitación del tratamiento para pacientes latinos – Parte 2

Existen diversos factores que desaniman a los latinos a la hora de buscar ayuda para trastornos de salud mental o por consumo de sustancias. Las preguntas de esta encuesta abordan las barreras y los facilitadores para los pacientes latinos que buscan ayuda para esos trastornos. Responda a las preguntas lo mejor que pueda.

* 15. * IDIOMA: los recursos que se enumeran a continuación podrían ayudar a los latinos a superar las barreras lingüísticas para obtener ayuda. Marque los que su clínica ofrece. Si conoce otras formas en las que su clínica ayuda a los latinos a superar las barreras lingüísticas, enumérelas en el espacio marcado como "comentarios".

- [ ] Traductor para hispanohablantes
- [ ] Clínicos que hablen español
- [ ] Señalización o folletos en español
- [ ] Telesalud en español para tratamiento virtual
- [ ] Ninguna de las anteriores

Comentario: si hay otras formas en las que su organización facilita el uso de servicios para los latinos a través de recursos lingüísticos, detállelas a continuación.

* 16. * AYUDA LEGAL Y FINANCIERA: los recursos que se enumeran a continuación podrían ayudar a los latinos a superar las barreras legales o financieras para obtener ayuda. Marque los que ofrece su clínica. Si conoce otras formas en las que su clínica ayuda a los latinos a superar las barreras legales o financieras, enumérelas en el espacio marcado como "comentarios".

- [ ] Una lista de números de teléfono de ayuda legal en español
- [ ] Un documento o folleto en español sobre los derechos de los latinos indocumentados
- [ ] Instrucciones en español para ayudar a los pacientes latinos a acceder a la cobertura para servicios
- [ ] Ninguna de las anteriores

Comentario: si hay otras formas en las que su organización facilita el uso de servicios para los latinos mediante recursos legales o financieros, detállelas a continuación.

* 17. CULTURA: los recursos que se enumeran a continuación podrían ayudar a los latinos a superar las barreras culturales para obtener ayuda. Marque los que su clínica ofrece. Si conoce otras formas en las que su clínica ayuda a los latinos a superar las barreras culturales, enumérelas en el espacio marcado como "comentarios".

- [ ] Admisión realizada por una persona latina/hispana
- [ ] Emparejamiento de pacientes latinos con personal latino
- [ ] Un entorno que refleje las culturas latinas
- [ ] Una evaluación relevante para la cultura
- [ ] Enmarcación de los problemas en un contexto cultural por parte del personal
- [ ] Uso de un modelo de intervención culturalmente específico por el personal
- [ ] Servicios de prevención culturalmente informados
- [ ] Servicios breves culturalmente informados
- [ ] Servicios infantiles de habla hispana en clínicas para niños latinos cuyos padres asisten a tratamiento
- [ ] Servicios comunitarios culturalmente informados
- [ ] Servicios de pareja culturalmente informados
- [ ] Servicios de crisis culturalmente informados
- [ ] Servicios educativos culturalmente informados
- [ ] Servicios individuales culturalmente informados
- [ ] Uso de ayudantes naturales o un enfoque sistémico
- [ ] Servicios de divulgación culturalmente informados
- [ ] Servicios de enlace de recursos
- [ ] La organización está dirigida por la comunidad latina
- [ ] La organización utiliza las instalaciones existentes de la comunidad minoritaria
- [ ] La organización tiene vínculos con la comunidad minoritaria
- [ ] La organización recurre a un defensor de los servicios de la comunidad latina
- [ ] La organización recurre a una o varias personas de la comunidad latina como asesor
- [ ] La organización recurre a una o varias personas de la comunidad latina como evaluador
- [ ] Una lista de números de teléfono de los recursos del vecindario para acoger a los latinos, como el Equipo de Extensión Comunitaria
- [ ] Alcance organizativo a las iglesias locales a las que puedan asistir los latinos
- [ ] Ninguna de las anteriores

Comentario: si hay otras formas en las que su organización facilita el uso de servicios para los latinos a través de recursos culturalmente sensibles, detállelos a continuación

* 18. ACCESO: los recursos que se enumeran a continuación podrían ayudar a los latinos a superar las barreras de acceso para obtener ayuda. Marque los que su organización (incluido su consultorio privado, si lo tiene) ofrece. Si conoce otras formas en las que su clínica ayuda a los latinos a superar las barreras de acceso, enumérelas en el espacio marcado como "comentarios".

- [ ] Recursos de telesalud en español para aquellos latinos que quieran tratamiento y no puedan acudir a la clínica por alguna dificultad
- [ ] Horarios de atención tardíos para las personas que trabajan a tiempo completo e intentan recibir tratamiento
- [ ] Transporte proporcionado para llegar a la clínica a aquellos que no cuentan con uno.
- [ ] Ubicación de la organización en una comunidad latina
- [ ] Fácil acceso para los latinos (en rutas de autobús o metro, o fácil disponibilidad de aparcamiento)
- [ ] Horarios/citas/visitas a domicilio flexibles
- [ ] La organización ofrece tratamiento inmediato (en un día o una semana)
- [ ] Ninguno de las anteriores
- [ ] Comentario: si hay otras formas en las que su organización facilita el uso de servicios para los latinos a través de los recursos de acceso, detállelas a continuación

* 19. OPIÁCEOS: los recursos que se enumeran a continuación podrían ayudar a los latinos a superar las barreras de acceso para obtener ayuda específica para los TCS relacionados con los opiáceos. Marque los que su organización (incluido su consultorio privado, si lo tiene) ofrece. Si conoce otras formas en las que su clínica ayuda a los latinos a superar estas barreras, enumérelas en el espacio marcado como "comentarios".

- [ ] Tratamiento asistido con medicamentos
- [ ] Médico in situ que puede recetar suboxone
- [ ] Mejora de la Recuperación Mediante la Consciencia Plena (Mindfulness-Oriented Recovery Enhancement – M.O.R.E.) para el abuso de opioides (por EL Garland)
- [ ] Derivación para tratamiento concurrente de trastorno por consumo de opiáceos
- [ ] Ninguno de las anteriores

Comentario: si hay otras formas en las que su organización facilita el uso de servicios para los latinos a través de recursos específicos para opiáceos, detállelas a continuación

* 20. ¿De qué otra manera su programa facilita (incluido su consultorio privado, si lo tiene) a los clientes hispanos/latinos o de habla hispana el acceso a los servicios? Explique en pocas frases en el espacio de abajo.

2026-6_W_TCCentrada_es

CARACTERÍSTICAS de su PRÁCTICA CLÍNICA – Parte 3

Bríndenos esta importante información sobre usted y su práctica clínica. Responda lo mejor que pueda.

* 21. ¿Cuál es su género?

- ◯ Femenino
- ◯ Masculino
- ◯ Otro

* 22. ¿Cuál es su edad? Introduzca un número entero (un dígito, no una palabra) en la casilla de abajo.

[ ]

* 23. ¿Con qué origen étnico se identifica?

- ◯ Negro/afroestadounidense
- ◯ Hispano/latino/descendencia española
- ◯ Asiático o del sur de Asia
- ◯ Nativo de Hawái u otras islas del Pacífico
- ◯ Caucásico no hispano/latino/descendiente española
- ◯ Nativo americano o nativo de Alaska
- ◯ Dos o más de los anteriores
- ◯ Otros

* 24. ¿Cuál es el nivel de estudios más alto que ha completado?

- ◯ Secundaria completa
- ◯ Grado técnico
- ◯ Bachiller (pregrado)
- ◯ Grado de maestría
- ◯ Doctorado

2026-6_W_TCCentrada_es

Superar las barreras al compromiso - Parte 5

Las preguntas en esta página simplemente solicitan que asegure que entiende el proceso de obtener su certificado y le solicitan que nos proporcione potencial

información de contacto de las personas que pudieran beneficiarse de esta capacitación.

* 25. Confirme que entiende que uno de los requisitos para obtener su certificado es la "asistencia", como en cualquier curso "presencial". Su presencia virtual será anotada y registrada.

- ◯ Sí
- ◯ No

26. Si conoce a otras personas que podrían beneficiarse de la capacitación gratuita en TCC que se ofrece en inglés y español, infórmeles al respecto. Estaremos encantados de ponernos en contacto con ellas si nos facilita su información de contacto en el espacio siguiente.

2026-6_W_TCCentrada_es

¡Felicitaciones! Vamos al siguiente paso.

Muchas gracias por elegir recibir esta capacitación en Terapia Cognitivo-Conductual Esencial para los Trastornos por Consumo de Sustancias. Ha completado la prueba de entrada. Su información nos ayudará a mejorar los servicios para otros clínicos de TCS. Haga clic en el botón Hecho (Done) para comenzar el curso. Comuníquese con Ruth Campbell a ruth@connectionscounseling.org si tiene alguna pregunta, o llámela al 845-255-5022.
