# Supplementary material for: Left Out and At Risk: Post-Pandemic Continuation of Organizational Service Reduction in Metropolitan New York City Coincides with Rise in Opiate Use and Mental Health Problems for Latinos
Source: Int J Environ Res Public Health. 2026 May 8;23(5):628. doi: 10.3390/ijerph23050628 (PMC13206180; doi:10.3390/ijerph23050628)
Supplement: Supplementary file 1 [file ijerph-23-00628-s001.zip › File S3 Provider survey for qualitative comments.pdf]

Provider questionnaire #3

## Welcome: Behavioral Health Provider questionnaire

**Thank you for taking the time to answer these questions concerning your practice. We're trying to see how services can be improved for Latinos and to counteract the toll on behavioral health services. The de-identified answers will be used in current and future research supported by Connections Counseling PLLC. Any personal answers you give will be kept confidential to the full extent of the law. The research questions have been approved by the North Star IRB, reachable at info@northstarreviewboard.org. No other organization has participated in designing nor has any other organization approved this research effort. You are not obligated to complete this questionnaire. Completed questionnaires will be entered into a drawing for one of ten $100 prizes. If your answers are drawn in the drawing, you will receive $100 as a check made out to you and mailed to your address or as a Zelle or Venmo transfer. The drawing will happen on November 21 at 9 pm. The average time to complete the survey is 5-7 minutes. The selected answers (with anonymous attribution to a "behavioral health provider in New York State") will be posted in any publication which is based on the collected data. Your feedback is very important in order to help us improve clinical services. Those who complete the survey will be regarded as having signed informed consent.**

* 1. Please enter your email address so we can communicate with you concerning your status in the drawing.

* 2. Please confirm your email address below.

Provider questionnaire #3

## Questionnaire concerning improving behavioral health services for Latinos

**Some say that programs that offer behavioral health (addiction and mental health) treatment for Latinos must seek to be culturally relevant in this time of moral panic and division. How should programs that want to offer behavioral health treatment to Latinos seek to become more culturally relevant?**

* 3. What might a program that is culturally relevant for Latinos be like? (Bilingual educational groups for the family member not in treatment? bi-lingual childcare for Latino children? telehealth? No telehealth? Is time of day important? Is anonymity important? Is transportation important?) Please express your ideas with a few sentences.

* 4. Many programs have stopped offering Spanish language lists of insurance that may help to cover treatment. Do you think that offering Spanish language information is useful to engage the Latino community? Why or why not?

* 5. Please add any other comments about how you believe behavioral health programs can improve the behavioral health (mental health and substance use disorder) treatment in the Latino community.

* 6. Practice Type (Which one most accurately describes your work setting?)

- ◯ Private or Outpatient Practice
- ◯ Intensive Outpatient/ Inpatient or Residential Practice
- ◯ Other (please specify)

* 7. If you practice in New York State, in what NY county do you practice? (If more than one, choose the one in which you practice most of the time. If you no longer practice in NY, please choose the first response - "I do not or no longer practice in New York State.")

* 8. Did you see clients in the past two weeks (for intake, assessment, in groups/families or individually)?

- ◯ Yes
- ◯ No

* 9. How many people are on your caseload? (If you have no clients, please put NA in the box below. Otherwise, please enter a number.)

* 10. Of the people that are on your caseload, how many are Latino? (If you have no clients, please put NA in the box below. Otherwise, please enter a number.)

* 11. Did the percentage of Latinos on your caseload change since last year?

- ◯ Yes
- ◯ No
- ◯ If the percentage changed, did it increase or decrease?

Provider questionnaire #3

## Congratulations! - here's the next step

**Thank you so much for choosing to answer this questionnaire. Your information will help us improve services for other clinicians. Please contact Ruth Campbell at ruth@connectionscounseling.org with any questions. Or call her at 845-255-5022. If your answers are chosen in the drawing, Ruth will contact you through the email that you have provided. Ten people will be chosen to get a $100 prize on November 21 at 9 pm. Note: if you participated in a drawing in September and have won a prize, you will not qualify to enter the drawing in November.**
